# International Interprofessional Collaborative Office Rounds (iiCOR): Addressing Children's Developmental, Behavioral, and Emotional Health Using Distance Technology

**DOI:** 10.3389/fpubh.2021.657780

**Published:** 2021-05-12

**Authors:** Jennifer S. H. Kiing, Heidi M. Feldman, Chris Ladish, Roopa Srinivasan, Craig L. Donnelly, Shang Chee Chong, Carol C. Weitzman

**Affiliations:** ^1^Child Development Unit, Khoo Teck Puat-National University Children's Medical Institute, National University Health, Singapore, Singapore; ^2^Department of Paediatrics, Yong Loo Lin School of Medicine, National University of Singapore, Singapore, Singapore; ^3^Department of Pediatrics, Stanford University, Stanford, CA, United States; ^4^Neurobehavioral Medicine Services, Mary Bridge Children's Hospital and Health Network, Tacoma, WA, United States; ^5^Developmental Pediatrics Unit, Ummeed Child Development Center, Mumbai, India; ^6^Department of Psychiatry, Dartmouth-Hitchock Medical Center, Lebanon, NH, United States; ^7^Division of Developmental Medicine, Boston Children's Hospital, Boston, MA, United States; ^8^Pediatrics and Child Study Centre, Yale School of Medicine, New Haven, CT, United States

**Keywords:** continuing education, interprofessional education, case-based discussion, interdisciplinary, international, mental health, developmental medicine, children

## Abstract

Developmental, behavioral, and emotional issues are highly prevalent among children across the globe. Among children living in low- and middle-income countries, these conditions are leading contributors to the global burden of disease. A lack of skilled professionals limits developmental and mental health care services to affected children globally. Collaborative Office Rounds are interprofessional groups that meet regularly to discuss actual cases from the participants' practices using a non-hierarchical, peer-mentoring approach. In 2017, International Interprofessional Collaborative Office Rounds was launched with several goals: to improve the knowledge and skills of practicing child health professionals in high and low resourced settings regarding developmental and mental health care, to support trainees and clinicians in caring for these children, and to promote best practice in diagnosis and management of these conditions. Five nodes, each comprised of 3–4 different sites with an interprofessional team, from 8 countries in North America, Africa, Asia, and South America met monthly via videoconferencing. This report describes and evaluates the first 2 years' experience. Baseline surveys from participants (*N* = 141) found that 13 disciplines were represented. Qualitative analysis of 51 discussed cases, revealed that all cases were highly complex. More than half of the cases (*N* = 26) discussed children with autism or traits of autism and almost all (*N* = 49) had three or more themes discussed. Frequently occurring themes included social determinants of health (*N* = 31), psychiatric co-morbidity (*N* = 31), aggression and self-injury (*N* = 25), differences with the healthcare provider (*N* = 17), cultural variation in accepting diagnosis or treatment (*N* = 19), and guidance on gender and sexuality issues (*N* = 8). Participants generally sought recommendations on next steps in clinical care or management. A survey of participants after year 1 (*N* = 47) revealed that 87% (*N* = 41) had expectations that were completely or mostly met by the program. Our experience of regular meetings of interprofessional groups from different countries using distance-learning technology allowed participants to share on overlapping challenges, meet continuing educational needs while learning about different approaches in high- and low-resourced settings. International Interprofessional Collaborative Office Rounds may prove a useful strategy for increasing the work force capacity for addressing developmental, behavioral, and emotional conditions worldwide. More systematic studies are needed.

## Introduction

Developmental, behavioral, and emotional disorders and disabilities [DBE disorders] are highly prevalent throughout the world. In the US, approximately one in 6 (17.4%) children aged 2–8 years has a diagnosis of a DBE disorder (1). Globally, of the more than 2.5 billion individuals under age 18, ~52.9 million children under the age of 5 years are estimated to have a DBE condition (2) of whom about 95% live in low- and middle-income countries. These numbers are likely to rise as millions of people continue to be displaced annually by climate change (3) and wars (4). Regardless of country of residence, interprofessional, coordinated healthcare, closely aligned with educational and social services, is essential to achieve good outcomes for the children and their families (5). Currently, health care systems around the globe are poorly resourced to address the needs of children with DBE disorders and their families.

At the heart of the inadequate health care response to children with DBE disorders is the lack of skilled child health professionals from different disciplines to diagnose and manage these children (6). Within high-income countries, disparities of care are worsening in many areas, including inner city and rural areas; regions that serve poor and marginalized communities remain underserved (7). Within low- and middle-income countries, the lack of qualified health care professionals negatively affects health care resources and delivery (8). In the absence of formal training, health professionals in low- and middle-income countries are typically either self-trained or trained in high income countries. Working in their native countries, they have few opportunities for peer learning or reflection due to a lack of colleagues and limited continuing education opportunities (9).

The World Health Organization (WHO) strongly advocates for an interprofessional approach to “mitigate the global health workforce crisis,” thereby strengthening existing healthcare systems and improving health outcomes (5, 10). The WHO defines interprofessional collaboration as “multiple health workers from different professional backgrounds working together with patients, families, carers (caregivers), and communities to deliver the highest quality of care” (5). Interprofessional collaboration has been found to improve professional and staff relationships (11, 12), enhance job satisfaction (10) and improve patient outcomes and satisfaction (13). In the US, Collaborative Office Rounds (COR), funded by the US Health Resources and Services Administration's Maternal Child Health Bureau (HRSA-MCHB), has been a model of interprofessional education since 1988 (14–16). Originally, COR groups were jointly led by developmental behavioral pediatricians and child psychiatrists and were targeted to increasing the capacity of general pediatricians to handle DBE issues. The groups met in-person at set intervals over sustained periods of time with a stable interprofessional membership. Discussions focused on cases from the clinical practices of team members in attendance. Evidence of the success of the model is that many of the original COR groups, such as the group in Michigan (17), are still meeting regularly after more than 25 years and have seeded other COR group-format meetings to expand the model of collaboration in their regions. In addition, 10 currently funded projects are expanding the format of COR, with increasing use of distance technologies.

In 2011, Dr. Weitzman from Yale University, in collaboration with Dr. Feldman from Stanford University, and Dr. Kiing and Dr. Chong from National University Hospital of Singapore, adapted the COR model for an international interprofessional collaboration. International Collaborative Office Rounds (ICOR) was funded by HRSA-MCHB. The ICOR model utilized monthly case-based discussions via online distance learning technology to allow peer-to-peer supervision. The cases were actual children and families from the participants' practices, predominantly those that challenged the clinicians. The discussions often included dilemmas or conflicts and sharing of the internal experiences of the clinician. Discussions also focused on balancing the provision of scientifically motivated care with patient or family needs and preferences, and the challenges of managing networks of health care, education, and social service.

In 2017, based on the success of ICOR, with a new grant from HRSA-MCHB and additional funding and technical support from the Society for Developmental-Behavioral Pediatrics (SDBP), the model was further adapted to reach a larger international audience and was renamed SDBP International Interprofessional Collaborative Office Rounds, Second Version or “iiCOR.” iiCOR expanded this online educational program of monthly interprofessional team meetings to many other countries and participants. iiCOR has provided a peer group venue for seasoned professionals, trainees, and professionals early in their careers who diagnose and manage DBE disorders across the globe. iiCOR was not only a rich opportunity for cultural exchange but when the covid-19 pandemic disrupted clinical care and in-service education around the globe, the online interprofessional in-service training became more relevant to participants in the face of travel restrictions and social distancing (18).

This study describes the 2-year experience and evaluation of iiCOR. The goals of this report are to describe how iiCOR was conducted, the range of cases discussed and evaluation of participating health professionals. We sought to determine the breadth of participation, the nature of the case discussion, and the satisfaction of participants.

## Methodology

### iiCOR Model

In 2017, teams from around the world were invited to apply to become an iiCOR site. Requirements included having an interprofessional team of at least 5 professionals, comprised of a combination of developmental-behavioral pediatricians, psychiatrists, general pediatricians, psychologists, and other child health professionals, who could meet monthly, ideally in one location, at a designated time for up to 90 min. Sites needed to assure that they had the necessary hardware and software to participate in online discussions through common video platforms. Sites indicated the day and time that their site members would be available for a group discussion and, to the extent possible, free of clinical responsibilities. Based on their application and their available meeting times, the organizing team combined sites into nodes, comprised of 3–5 sites. Each node included a mix of sites from high- and middle- and/or low-income countries.

The project was designed to use a non-hierarchical, peer supervision model to encourage learning through presentation of actual cases from the participants' clinical practices. In addition to diagnosis and management, the model's focus on reflection was designed to encourage attention to all of the relationships involved in clinical care, including the relationships between parents and patients; clinicians, patients and families; and moderators and clinicians. Each site designated a moderator and co-moderator. The moderator's role was to manage the discussion, such that many different voices and disciplines could contribute, and that tone remained respectful. The moderator was also primed to assure patient confidentiality was maintained, and the discussion remained true to the presenter's key questions. Prior to the launch of the program, the moderators participated in a training, which reviewed the goals of iiCOR, techniques for encouraging all participants to contribute, and information for troubleshooting for technical difficulties. Initial iiCOR sessions were attended by Drs. Weitzman, Feldman and Kiing to assure fidelity to the model.

Each month, one of the sites within the node assumed responsibility for the case presentation of that session. No specific instructions were provided regarding the nature of the case beyond the requirement that (1) it was an actual case from one of the clinician practices, (2) it posed a challenge to the clinicians, and (3) it included specific questions for the group. Participants understood to strip case presentations of any personal health information. In preparation for the discussion, the host site posted a de-identified summary of the case to a password protected website, accessible to all and only iiCOR participants, with at least one question for the group to consider in preparing for the discussion. SDBP administrative staff sent out Zoom invitations to participants in the week prior to the monthly discussion and monitored the website. At the time of the discussion, the host site moderator served as facilitator for the discussions. Within the nodes, sites rotated leading discussions.

### Evaluation Strategies and Analyses

Description and evaluation of the program results did not require IRB approval as it did not meet the criteria for human subject's research given that there are no patient contacts and/or personal health identifiers.

A baseline enrollment survey was completed prior to the program launch to describe the diversity of participants. This survey included questions about the participants discipline, years in and type of practice, whether they were a trainee, experience with COR, country of origin and reasons for participation. We analyzed the nodes and their members using simple descriptive statistics.

To understand the challenges of the clinicians who enrolled in this program, we reviewed and analyzed the cases presented. The source of the analysis were the written descriptions submitted to the website prior to the discussion, including key questions, and the summaries prepared by the presenters at the conclusion of the discussion. There was no opportunity to analyze the actual discussions as the sessions were not recorded. Three coders who were iiCOR participants and co-authors identified the diagnosis of the child in the case and key themes that were listed in initial case presentation uploaded prior to the discussion and the summary form of the discussion. The coders assessed the “dominant” theme which emerged from the discussion and also “other” secondary themes. Cases were double-coded for themes and any disagreements among coders were discussed until consensus was reached. An audit trail was established of the data collection and analysis process.

At the conclusion of each iiCOR discussion, a member of the presenting site was asked to complete a short questionnaire on key learning points and cultural lessons gained as a result of the discussion with input from the group. We conducted an analysis of these summaries in which two coders who were iiCOR participants reviewed these key learning points and classified them with disagreements discussed until consensus was reached. We had also requested that participants complete a brief individual session survey comprising six questions at the conclusion of each case discussion regarding relevance of the discussion and participant satisfaction. However, these surveys were frequently not completed and so they were not analyzed in this evaluation.

At the 1-year point, participants were asked to complete a general satisfaction survey and impact the program had on their practice. The satisfaction survey was designed by the organizers specifically for this project. Simple descriptive statistics were used to describe results of the baseline and general satisfaction surveys completed. A qualitative descriptive approach was utilized to evaluate cases posted to the website to reveal key themes.

## Results

### Nodes, Sites, and Participants

In 2017, 22 teams applied to become sites. A total of 19 sites were organized into 5 nodes. The decision of which sites formed a node was primarily driven by two constraints: (1) time zone and (2) country. Understandably, teams chose to meet immediately before or after-work hours. Preferred day of the week varied by group. Thus, the number of arrangements was highly limited. The other consideration was that the nodes should have sites from high- and middle- or low-income countries. Fortunately, we were able to meet this requirement. Altogether, the sites represented 8 countries in Africa, Asia, South America and the US. Table 1 below describes the nodes, named by their members, the sites within each node, the time zone of the site, and the time that the node met.

**Table 1:** Nodes and Sites comprising the International Interprofessional Collaborative Office Rounds Program, including Time Zones for each of the sites.

**Node name**	**Sites**	**Time zone**
Champions	•Children's Hospital of Philadelphia, PA, USA •Rose F. Kennedy Centre at Children's Hospital at Montefiore, Bronx NY, USA •Lokmanya Tilak Medical College, Mumbai, INDIA •Birat Medical College and Teaching Hospital, Birat Nagar, NEPAL	GMT – 4:00 •Pennsylvania, USA •New York, USA GMT + 5:30 •Mumbai, INDIA GMT + 5:45 •Biratnagar, NEPAL
Sunshine	•University of California, SAN DIEGO, USA •Mary Bridge Children's Hospital and Health Network, Tacoma, WA, USA •Rwanda DBP Peds Group, Kigali, RWANDA •Combined Cape Town, Harare, ZIMBABWE & SOUTH AFRICA	GMT – 7:00 •San Diego, USA •Washington, USA GMT + 2:00 •Kigali, RWANDA •Harare, ZIMBABWE •Cape Town, SOUTH AFRICA
S-A	•Nationwide Children's Hospital, Columbus, OHIO, USA •Western Michigan University Homer Stryker MD School of Medicine, MICHIGAN,USA •Boston Medical Centre, Boston, MA •LIH Olivia's Place, Shanghai & Shenzhen, China	GMT – 4:00 •Ohio, USA •Michigan, USA •Massachusetts, USA GMT + 8:00 •Shanghai, CHINA
CHIPPS	•Case Western Reserve University, Cleveland, OHIO, USA Meyer •Center for Developmental Pediatrics—Texas Children's Hospital/Baylor College of Medicine, Houston, TEXAS, USA •The Children's Hospital & Institute of Child Health, Lahore, PAKISTAN •USIG-PMH (US/India-Pediatric mental health)	GMT – 4:00 •Ohio, USA GMT – 5:00 •Texas, USA GMT + 5:00 •Lahore, PAKISTAN •INDIA
IndyROCDart	•Children's Hospital at Dartmouth-Hitchcock, NEW HAMPSHIRE, USA •Riley Children's Health, INDIANAPOLIS,USA •Shenzhen Children's Hospital, Shenzhen, CHINA	GMT – 4:00 •New Hampshire, USA •Indianapolis, USA GMT + 8:00 •Shenzhen, CHINA

Baseline enrollment information was obtained from 141 participants. A total of 13 disciplines were represented; the most common were Developmental Behavioral Pediatrics (41.1%), Child and Adolescent Psychiatry (17.0%), General Pediatrics (17.0%), and Psychology (10.6%). The other disciplines (14.3%) included nursing, occupational therapy, public health, social work, speech-language pathology, law, business, social science, and genetics. Participants had a range of professional experience. Of the 141, 27 participants (19.1%) had over 20 years in practice, 23 (16.3%) had 16–20 years in practice, 20 (14.2%) had 11–15 years of experience, 23 (16.3%) had 6–10 years of experience, and 48 (34.0%) had <5 years in practice. Of these, 22 (15.6%) were trainees.

All five nodes continued to meet together for a 2-year period.

### Cases Discussed

The first 51 cases uploaded to the password-protected website portal were analyzed. Approximately half of the cases (*N* = 26) focused on individuals with autism and approximately half (*N* = 25) focused on individuals with intellectual or developmental disability. The median age of patients within the cases brought for discussion was 8.5 years (Range 4 months-28 years), and clinicians discussed transition to adulthood and adulthood in four cases (8%).

The cases discussed in iiCOR were highly complex. Very few cases discussed focused on diagnostic questions or uncertainties exclusively; all included aspects of management of multiple problems or issues, summarized as themes. The vast majority of case summaries (*N* = 49) contained three or more themes. Key themes identified in discussions, examples of the theme and case counts are found in Table 2. The most frequent themes were family dynamics/social determinants of health (19) (*N* = 31) and the child's aggression to others or to self (*N* = 25). Another frequent theme was differences of opinion either within the family or between the family and the clinicians (*N* = 17).

**Table 2:** Themes from case descriptions.

**Themes**	**Topics included in the themes**	**Dominant theme (number cases)**	**Additional themes (number cases)**
Family dynamics and social determinants of health	Domestic violence, trauma, child neglect or abuse, parental divorce or separation, death in the family, family substance use, or family poverty	10	31
Aggression and Self Injury	Physical or verbal aggression to family members, peers, school community or self	10	25
Differences with the health provider	Disagreements between the family and health care provider regarding prescribed therapies or intervention, difference in expectations for child between parents and clinicians	8	17
Gender and Sexuality	Gender dysphoria, gender identity or assignment, puberty, sexuality	6	8
Psychiatric co-morbidity	Diagnosis and management of mood and anxiety disorders, suicidality, hallucinations, and delusions	5	31
Medically Complex Care	Congenital, chromosomal or genetic (e.g. Down syndrome), complex congenital heart disease	5	18
Cultural differences	Discussion of values, practices, beliefs, customs, attitudes of the patient, family and/or health professional	3	19
Transition to Adulthood	Issues related to becoming an adult	2	4
ASD and ASD like features	Core features and associated symptoms	1	26
Dev delay/ID/regression	Core features and associated symptoms, developmental regression	0	25
Sleep	Sleep disruptions, behavioral sleep issues, biological causes of sleep disturbance	0	8
ADHD	Core features and associated symptoms, psychosocial and educational issues	1	12

### Session Summaries

Over half of the 51 cases (*N* = 29, 56.8%) had a session summary completed shortly after the discussion. The moderator or another member of the presenting was asked about key learning points at the completion of each session. These key learning points were categorized into topics and summarized in Table 3. The most frequently cited key learning point was “culture and site-specific practice variations” (*N* = 15) followed by “diagnostic challenges and uncertainties” (*N* = 13). Practice variation revealed rich discussions around differences in resources, school systems and legal systems around the world. Participants found that differences in culture, cultural practices and beliefs, as well as cultural-bound approaches to care between the sites within each node, provided some of the most interesting, enriching and rewarding discussions. Cultural variations impacted practice and learning in many ways—from the effect on the family's ability to seek help, to the ease of engaging families in healthcare systems, to the family's use of alternative therapies and treatments. Cultural discussions frequently cut across most learning themes.

**Table 3:** Learners' self-descriptions of the key learning points of the sessions and their related case examples.

**Key learning points**	**Examples**
Managing diagnostic challenges or uncertainties	A child with suspected neurodevelopmental problems and experiencing parental separation presented with socially inappropriate behaviors and emotional outbursts. Discussion raised questions about multiple diagnostic possibilities including adjustment disorder, autism, and/or anxiety diagnosis.
Exploring cultural and practice variations across the world, and differences in medical treatment	A Nepalese child with acute psychosis and depression had a challenging home environment. Discussion explored the possibility of child abuse and contrasted the definition of “abuse” and availability and nature of child protection systems across cultures and countries.
Supporting children and families with complex needs	A transgender adolescent with mental health issues feared abandonment by the family while parents were struggling with the diagnosis and needs of their child. Discussion highlighted strategies for supporting families whose children have complex care needs.
Providing family-centred care	A child with ADHD and challenging behaviors lived in a kinship adoptive family. Previous recommendations for medications and behavioral treatment had not been followed. Discussion highlighted the importance of understanding the complex interplay of stigma, perception, and parental beliefs.
Addressing complex mental health issues	A Chinese adolescent with ADHD developed comorbid obsessions and started to hear voices. A diagnosis of schizophrenia, personality disintegration, obsessive compulsive disorder was made. Discussion focused on using antipsychotics to bring symptoms under control, managing side effects of medication and working with the family.
Recognizing the importance of family engagement in management	Parents of a child with autism divorced between initial evaluation and follow up. One parent wanted the “label” removed while the other parent wanted to enroll the child in intensive autism services. Discussion highlighted the risks of marital discord in children with neurodevelopmental disorders, and ways to re-engage adversarial families.
Understanding psycho-education and psychotherapy	An adolescent from rural San Diego experienced frequent mood swings. Discussion reviewed management and treatment issues, including the importance of psychoeducation for both the teen and the parent regarding mental health disorders.
Appreciating the need for multidisciplinary evaluations	A Spanish-speaking child in the US was diagnosed with selective mutism. Discussion focused on the need for a multidisciplinary assessment and collaboration with schools.
Appreciating the importance of continuity of care and reducing barriers to long term follow up	Parents of a child from rural Mumbai with possible Rett syndrome could not afford follow-up care and declined further treatment because of gender. Discussion highlighted barriers to care including; financial limitations, fatalistic attitudes, gender inequity, and global resource limitations.
Generating alternative treatments	Parents of a child with autism and extremely challenging behaviors wanted to explore the use of Medical marijuana in the child. Discussion focused on about evidence- and non-evidence based treatments, and complementary and alternative treatments used globally.

### Year 1 Evaluation

Forty-seven respondents completed the survey at the conclusion of the first year of participation. Overall satisfaction was scored using a Likert scale, with 1 equaling ‘highly unsatisfied’ and 4 indicating ‘high satisfaction’. The mean degree of satisfaction was 3.6; only one participant rated the sessions as poor. A total of 41 (87%) said their expectations were “completely” or “mostly” met by the program. A total of 39 (83%) of participants reported that iiCOR had some or definite impact on their practice as it related to DBE disorders in children and only one participant reported minimal or no impact.

Over the first 2 years of operation, all five nodes maintained the schedule of monthly meetings (with brief summer holiday breaks). Though not a formal element of the program, many sites shared resources that were pertinent to the case, including printed materials, rating scales, websites, and other information to support health care clinicians and families. In addition, participants shared a wide array of standardized assessment instruments that can be used in screening, assessment, and diagnosis, especially those in the public domain. Many sites also provided their attendees CME credit for attendance.

## Discussion

In summary, we are facing a global crisis of children experiencing developmental, behavioral and emotional disorders that can only be expected to increase in the future. However, the number of professionals trained to diagnose and manage these conditions is limited and cannot meet the growing needs. There are a range of potential solutions to address this discrepancy. This paper focuses on a relatively low-cost continuing education program designed to improve knowledge and skills of professionals who care for children with DBE disorders worldwide. Here, we describe how we successfully implemented an international, interprofessional case-based Collaborative Office Rounds program, designed to improve the knowledge and skills of professionals about developmental, behavioral, and emotional problems in children. The model created 5 nodes, comprised of 19 different sites from high-, middle-, and low-income countries. A total of 141 participants from 13 disciplines shared cases together in a non-hierarchical, reflective peer-supervision model. A key feature of the model was that the discussions were focused on actual cases that challenged the participating clinicians. We saw that the cases discussed were highly complex, with more than three distinct themes in almost every case. Participants expressed a high level of satisfaction with the program.

### Challenges in Implementing International Collaborations

While we do not have data to support this observation, one of the biggest challenges in developing an international program was finding times when people could meet given the wide differences in time zones. Members of the sites had to find times free from clinical or other responsibilities. They highly preferred to meet immediately before or after work hours, especially important when groups were meeting in person, rather than from home in the evening. This challenge may represent a barrier for widespread generalization. Changes from standard to daylight savings times and unanticipated national holidays further complicated scheduling meetings across the year. As a program like iiCOR seeks to expand, it will likely attract only the most motivated and organized sites across the globe. Creation of regional nodes may address this issue and reduce this barrier as there may be fewer time zones to traverse. Additionally, good internet connectivity is required to participate in a program such as iiCOR and the requirements were made clear when sites submitted an application to participate. This requirement again may limit the reach of another such a program in the future.

Each site was tasked with identifying a local team that works together on behalf of children with DBE disorders and their families. While most participants were from medical specialties, psychologists, and other health professionals, such as occupational therapists, social workers, and speech and language pathologists were also represented. Participants included highly experienced and relatively inexperienced clinicians. Sites successfully involved trainees, which provided an important early career exposure and opportunity to experience the benefit of international collaboration. Although many sites already had existing interprofessional relationships, one of the potential benefits from becoming an iiCOR site would be developing local relationships and contacts that may not have existed prior to participation.

Our data collection methods did not allow us to calculate the percentage of participants that continued to attend the meetings throughout the first years of the program. However, all of the nodes continued to meet together monthly for at least 2 years, though we know that some participants fell off over that time.

### Complexity of Cases

The cases which sites brought for discussion were highly complex, multifaceted, and challenging. This complexity likely arose because the participants were specialists and subspecialists, to whom the most challenging children and families would be referred. Such cases are often difficult to understand and manage without the input from colleagues and iiCOR provided the additional benefit of international colleagues. The participants may have also reasoned that such complex cases would pique the interest of the others within the node. The cases rarely involved as their primary question, diagnostic questions or dilemmas. Rather, the participants were often looking for recommendations on next steps in clinical care or managing their work in complex systems of care. The duration of the sessions, up to 90 min, allowed for an in-depth consideration of multiple issues. iiCOR cases also highlight the possibility that when any case involving DBE disorders is discussed from an interprofessional and international perspective the complexity of caring for these children and their families is illuminated.

A few of the major themes discussed in the groups warrant discussion. Participants frequently presented cases highlighting understanding and managing complex family and social circumstances. Professionals serving children with disability almost always interact with the families of their patients, who frequently rely on them to provide education, behavior management and, importantly, emotional support. Clinicians frequently presented cases describing challenges working with families, including families who sought multiple opinions from one professional to another in search of an unsubstantiated diagnosis; divorced parents who disagreed on the child's diagnosis; families who reacted strongly to a diagnosis when working through an interpreter; and parents who demanded that a diagnosis in the medical record be changed. iiCOR illuminated how culture, resources, and stigma affect addressing psychosocial issues across the globe. Case discussions further highlighted differences in the expected role of women as patients, family members, and health professionals. iiCOR also highlighted differences in the doctor-patient relationship across the global and the role of shared decision making. iiCOR discussions shed light on issues of racism, marginalization of specific groups, income disparity, and a lack of equity that transcended whether a country was a high, middle or low-income. These discussions highlighted that thinking about children with DBE disorders must be linked to evaluating the functioning and well-being of families, communities, and systems that surround the children if there is to be individual and global improvement in this crisis.

The cases presented highlighted the frequent co-occurrence of developmental disorders with mental health problems. Other critical issues such as gender and sexuality were themes of the discussion. Cross-cultural discussions highlighted unique cultural taboos and stigma. Discussions included the role of faith healers, religious, and alternative treatments.

### Interpersonal Aspects of iiCOR

iiCOR is distinguishable from other case-based discussion models in the departure from strictly didactic presentations in a medical model style. The approach here encouraged clinicians to discuss the interpersonal aspects of care, such as personal feelings related to patients and to their work. As the economics of medicine have infringed on the ability to discuss cases with the treatment team, these types of case discussions that emphasize non-hierarchical peer supervision have become an elusive luxury. Our implementation of iiCOR can be compared to the model of Balint groups (20–22) which were developed in the United Kingdom in the 1950s and were designed to allow clinicians to talk about their feelings and their work (including their mistakes) in a safe environment and to focus on the doctor-patient relationship. Studies of Balint groups have shown that they can reduce clinician burnout, promote greater work satisfaction and reduce professional loneliness and isolation (20). Balint groups tend to be intensive and frequent whereas iiCOR was less intensive and monthly. However, the model attempted to create a “safe” environment for clinicians to express vulnerabilities, emotional reactions to patients and colleagues and get feedback and support. Although the clinicians did not specifically mention the safe and accepting environment as a positive feature of the learning, their ability to present highly complex cases, especially those with complex family dimensions and intractable social determinants of health were an unburdening of the stresses of care for children with DBE conditions. It is important to recognize that this type of discussion model is not well-known and utilized, particularly in low- and middle-income countries. Participants from low- and middle-income countries reported that cases did not always reflect the typical issues seen in their developmental practices. Yet, the non-hierarchical nature of the discussions were highly valued particularly amongst the participants from low- and middle-income countries, where doctors as specialists would be expected to lead and direct case management. Additional evaluation is required to assess the efficacy and acceptability of this model globally.

### COVID-19

We would be remiss not to mention the tremendous value of a global consultation group occurring during a year fraught with a pandemic and substantial social and governmental unrest. As iiCOR participant meetings increased, participants developed relationships with their colleagues that expressed concern not only for the welfare of one another's patients but also for each other. It was noted that meetings often started with updates related to how participants were faring during the pandemic, how they and their families were coping, and how individual practice styles were being impacted. Zoom meetings that had initially taken place from office spaces began to shift to homes, adding to the personal nature of the connections being made. In short, during a time period when separation and isolation were greatly increased throughout the world, iiCOR participants were given a unique and welcomed global opportunity to build support and enduring connections.

### Limitations

Only 56.8% of cases had a session summary completed at the conclusion of the session and this limited our understanding of key learning points. Although we asked moderators to take attendance after each session, this was done inconsistently, and it was difficult to assess consistency of individual participation. Importantly, only 47 respondents completed the annual evaluation, which limits the interpretation of findings. We know that the respondents are likely to represent a biased sample. We did not assess practice change or challenges faced by our participants in our evaluations and were limited to obtaining key learning points, satisfaction ratings, and self-assessment of the impact of the program on practice. We think that the key to improving collection of forms and evaluations is the addition of funding and staff dedicated to the project, which was beyond the scope of this project. Of course, staff increases the expense of the program. Additional research in the future should focus on whether participation in iiCOR is able to change and improve clinical practice. A final limitation is that the discussions did not include patients or their families or group members whose role was specifically to cover the patient/family perspective. Such an effort would be interesting though challenging in future projects.

## Conclusions

As the crisis of children with developmental, behavioral, and emotional disorders around the globe increase, low cost interventions, such as iiCOR designed for the potential to improve workforce capacity and interprofessional collaboration will become increasingly critical. This paper demonstrates that distance-learning technology can be used for participants to meet their educational and professional needs, discuss complex cases, and explore cross-cultural and sensitive issues. This study demonstrated that developmental and child mental health professionals from different countries and different cultures face overlapping challenges irrespective of the wealth of a nation and learn from each other in monthly sessions. Lastly, iiCOR demonstrated that when given the opportunity and a specific learning climate, clinicians discuss cases with great complexity and share personal stories. iiCOR has the potential to excite, inspire and catalyze global collaborative care efforts among established experts and clinicians in the early stages of their careers. More systematic studies are needed to determine the impact on workforce capacity.

## Data Availability Statement

The original contributions presented in the study are included in the article/supplementary material, further inquiries can be directed to the corresponding author/s.

## Ethics Statement

Analysis of the program results did not require IRB approval as it did not meet the criteria for human subjects research given that there are no patient contacts and/or personal health identifiers.

## Author Contributions

CW, HF, JK, and SC developed the model. JK, HF, CL, RS, CD, SC, and CW made substantive revisions to the manuscript and participated in the program. JK, HF, and CW conceived and designed the data analysis. CW and HF collected the data. JK, HF, CW, CL, and SC performed analyses. JK and HF drafted the manuscript. All authors approved the final version.

## Conflict of Interest

The authors declare that the research was conducted in the absence of any commercial or financial relationships that could be construed as a potential conflict of interest.
